# Maternal Inflammation Alters Nuclear and Mitochondrial DNA Methylation Patterns in Neonatal Brain Monocytes

**DOI:** 10.3390/cells15080714

**Published:** 2026-04-18

**Authors:** Andrew T. Ebenezer, Jonathan R. Hicks, Brooke Hollander, Alexander Hone, Mona Batish, Robert Akins, Adam Marsh, Elizabeth Wright-Jin

**Affiliations:** 1 Division of Neurology, Nemours Children’s Hospital, Wilmington, DE 19803, USA; andrew.ebenezer@nemours.org (A.T.E.); brooke.hollander@nemours.org (B.H.); alexander.hone@nemours.org (A.H.); 2 Division of Biomedical Research, Nemours Children’s Health, Wilmington, DE 19803, USA; jonathan.hicks@nemours.org (J.R.H.); robert.akins@nemours.org (R.A.); 3 Sidney Kimmel Medical College, Thomas Jefferson University, Philadelphia, PA 19107, USA; 4 Medical and Molecular Sciences, University of Delaware, Newark, DE 19707, USA; batish@udel.edu; 5 Delaware Data Science Institute, Center for Bioinformatics and Computational Biology, University of Delaware, Newark, DE 19716, USA; amarsh@udel.edu; 6 School of Marine Science and Policy, University of Delaware, Newark, DE 19107, USA; 7 Psychological and Brain Sciences, University of Delaware, Newark, DE 19107, USA

**Keywords:** hypoxic ischemic encephalopathy, maternal immune activation, DNA methylation, epigenetics, neurodevelopment

## Abstract

**Highlights:**

**What are the main findings?**

- Widespread DNA methylation changes occur in brain monocytes of newborn mice after exposure to maternal immune activation in utero.
- Mitochondrial DNA is hypermethylated in offspring’s brain monocytes after exposure to maternal immune activation in utero.
- Nuclear genes are predominantly hypermethylated after maternal immune activation, and gene ontology analysis reveals these genes are important for neurodevelopment, immune response, and structural development.

**What are the implications of the main findings?**

- Altered methylation of nuclear and mitochondrial DNA in brain monocytes may increase neurodevelopmental risk in offspring after maternal immune activation in utero.

**Abstract:**

Neonatal hypoxic ischemic encephalopathy (HIE) is a common birth complication that can cause death or lifelong disabling conditions like cerebral palsy, epilepsy, and autism. It is well established that maternal infection and inflammation are significant risk factors for HIE but reasons for this increase in neurological risk to the offspring remain unknown. Inflammation or infection are associated with epigenetic changes and may contribute to the increased risk of neurodevelopmental disability in exposed offspring. Here, we analyzed and compared DNA methylation patterns in brain monocytes isolated from control, maternal immune activation (MIA), and an inflammation sensitized HIE (IS-HIE) CF-1 mouse model at postnatal day 7. We found that maternal inflammation induced significant methylation differences in neonates relative to control samples in both MIA and IS-HIE samples with no significant differences identified between the MIA and IS-HIE groups. MIA samples showed hypermethylation at loci involving craniofacial development and transcription factors important for regulating neurodevelopment and immune function. MIA samples also demonstrated significant hypermethylation at multiple mitochondrial genome CpGs. These findings suggest that maternal inflammation induces epigenetic alterations in fetal brain immune cells that are detectable in neonates. These changes may contribute to heightened neurodevelopmental risk in offspring following hypoxic injury, highlighting potential molecular pathways for future therapeutic targeting.

## 1. Introduction

Neonatal hypoxic ischemic encephalopathy (HIE) is a common neurologic condition caused by insufficient oxygen and blood flow to the brain around the time of birth [1]. Neonatal HIE occurs in approximately 1–3 per 1000 births in developed countries, with up to 15–20 times higher incidence in low- and middle-income countries [2,3]. Neonatal HIE can result in death, physical, and/or mental disabilities, such as cerebral palsy, epilepsy, developmental delay, intellectual disability, and autism [4]. Currently, therapeutic hypothermia is the only effective treatment for neonates with HIE [5]. However, this treatment is constrained by a narrow window, only 6 h after birth, in which the therapy must be initiated [6]. Furthermore, it has proven ineffective in low- and middle-income countries, where the incidence of neonatal HIE is highest [7].

Maternal immune activation (MIA) resulting from infection or inflammation is a major risk factor for neonatal HIE. MIA increases the likelihood of diagnosis of HIE by up to 8-fold [8,9,10,11,12]. In addition, preceding MIA increases the risk of neurodevelopmental deficits. For example, the risk of cerebral palsy increases by 4.7-fold in neonates with HIE where there is preceding inflammation or infection [8,9,13,14,15,16]. The mechanisms underlying this increased neurologic risk after MIA remain poorly understood, but recent studies implicate epigenetic pathways. Chorioamnionitis, an intraamniotic infection, has been associated with DNA methylation changes in the cord blood of human offspring [17], and stable methylation changes have been found in both peripheral mononuclear cells and skeletal muscle of children with cerebral palsy [18,19]. These findings implicate DNA methylation in neurologic risk and suggest that widespread gene expression changes may be occurring. In a mouse model of inflammation sensitized HIE (IS-HIE), our group previously identified upregulation of epigenetic regulatory pathways infiltrating brain macrophages [20]. Intrinsic microglia and infiltrating macrophages are the primary initial responders to brain insults such as hypoxia or inflammation and direct the downstream response to injury largely via phagocytosis and recruitment of additional inflammatory cells [21]. Given these findings, we hypothesized that exposure to in utero inflammation increases neurologic risk due to functional immunological changes in the offspring driven by epigenetic changes in brain monocytes.

Epigenetics describe the regulatory process by which gene transcriptional activity is enhanced or inhibited without changing the underlying DNA sequence. Epigenetic mechanisms of gene transcription regulation include histone modifications, DNA methylation, and RNA interference. Histone modifications chemically alter the histone proteins involved in chromatin, altering access to DNA sequences. DNA methylation occurs when a methyl group is covalently added to nucleotides by DNA methyltransferases (DMNTs). Some of these DNA methylation patterns can be lasting and heritable across generations, however, most methylation events are transient phenomenon, particularly those involved in regulating neurodevelopment in response to environmental stressors [22].

In this study, we used a previously developed model of HIE that combines MIA in utero with a deep global hypoxia in mouse pups at postnatal day 6 (P6) with isolation of brain monocytes at P7 [20]. Maternal lipopolysaccharide (LPS) exposure mimics inflammatory signals such as those that occur in chorioamnionitis, a common perinatal condition that predisposes to HIE [10]. MIA exposure preceding hypoxia creates similar pathologic conditions as would be experienced in a majority of pregnancies resulting in HIE diagnosis [23]. This approach allowed us to investigate the epigenetic mechanisms impacted by exposure to intrauterine inflammation.

## 2. Materials and Methods

Animals. CF-1 (Charles River International Laboratories, Malvern, PA, USA) timed pregnant dams and their offspring were used. Mice had a 12-h light-dark cycle with free access to food and water. The Nemours Institutional Animal Care and Use Committee approved all procedures (RSP21-27351-002).

Study design. Experimental groups included maternal immune activation (MIA), inflammation sensitized HIE (IS-HIE). The control group was exposed to maternal saline injection and normoxia. All cells isolated from an individual mouse brain were considered an experimental unit. Four experimental units with equal males and females were allocated to each group. The total number of animals used in this study was 12. Sample size was not determined a priori. All animals and data points were included in analysis. Animals in the control group were from a single litter. Animals in the MIA and IS-HIE group were from a single litter. No inclusion or exclusion criteria were set a priori. There were no excluded animals or data. Confounders such as the order of treatments or measurements and animal cage location were not controlled. Outcome measure was differences in methylation.

Maternal immune activation. Timed pregnant dams were exposed to low dose lipopolysaccharide (LPS) as previously described [20]. Briefly, dams were given 50 ug/kg LPS or 0.05 mL 0.9% saline via intraperitoneal injection at embryonic day 18.5 (E18.5).

Hypoxia exposure. Mice were subjected to deep global hypoxia on P6 as previously described [20]. Briefly, mice were subjected to 8 min of progressive hypoxia from 21% to 0% oxygen or normoxia (21% oxygen) using a hypoxia chamber (Biospheryx, Parish, NY, USA).

Cell dissociation. Whole brains were collected from male and female pups on P7 and dissociated into a single cell solution using the Adult Brain Dissociation Kit and gentleMACS Octo Dissociator (Miltenyi Biotec, Gaithersburg, MD, USA) according to the manufacturer protocol.

Monocyte isolation. Brain monocytes were enriched via magnetic CD11b-coated beads (Miltenyi Biotec, Gaithersburg, MD, USA) according to manufacturer protocol. Cell pellets were flash frozen and stored at −80 °C until genomic DNA extraction.

Methylation sequencing. Genomic DNA was extracted using the DNeasy Blood & Tissue Kit (QIAGEN, Germantown, MD, USA). Infinium Mouse Methylation Bead Chip Assay (Illumina, San Diego, CA, USA) was performed by CD Genomics (Shirley, NY, USA).

Methylation analysis. Methylation loads were provided by CD Genomics following a standard pipeline as β values. Quality control, methylation calling, statistical analyses and visualization were performed using a standardized pipeline conducted in R version 4.5.0 (R Core Team). The data was merged with the Mouse Infinium Methylation BeadChip manifest file obtained from CD Genomics to ensure accurate annotation and genomic alignment of CpG sites for downstream analyses. Statistical testing was conducted using minfi (version 1.56.0) [24], limma (version 3.66.0) [25], dplyr (version 1.1.4) [26], tidyverse (version 2.0.0) [27], openxlsx (version 4.2.8.1) [28], readr (version 2.1.6) [29], ENmix (version 1.46.0) [30], and GenomicRanges (version 1.62.1) [31], visualizations utilized ggplot2 (version 4.0.1) [32], circlize (version 0.4.17) [33], geneplotter (version 1.88.0) [34], treemap (version 2.4.4) [35], colorspace (version 2.1.2) patchwork (version 1.3.2), and sesame (version 3.9) [24,25,31,33,36,37]. Annotation was handled by annotatr (version 1.36.0) [38], biomaRt (version 2.66.0) via Ensembl [39].

Principal Component Analysis was performed in R and plotted with *ggplot2* [32,40], to plot the largest sources of variation in the methylation profiles.

M = Log2((β+ε)/(1−β+ε)),(1)

EQ1: Equation describing the conversion from beta values of methylation to M values.

β values were logit transformed to M-values using EQ1 [41] where ε was set to 1 × 10^−6^ to account for infinities generated in instances of complete methylation or complete demethylation. The samples were assigned into three treatment groups “HIE”, “MIA” and “Control”. We then performed linear modeling and empirical bayes calculation for differential methylation [42]. Benjamini-Hochberg False-Discovery-Rate correction was then performed, identifying CpG loci that are differentially methylated. Data, including false-discovery-rate-corrected and uncorrected *p*-values of differential methylation were imported and processed with dplyr. Volcano Plots were generated using ggplot2 and limma to visualize the statistical significance of the CpGs, displaying log_2_ fold changes against –log_10_(*p*-values).

Circos plots were generated to visualize the genome-wide methylation changes and their contexts. We assessed CpGs that fall within the gene, or up to 2000 base pairs upstream of the transcription start site to capture promotor regulatory CpGs that may influence transcription. Genomic overlaps were identified between differentially methylated CpGs and protein-coding genes in BiomaRt. A Circos plot was generated to show differences in methylation β values of all CpGs, listing genes with differentially methylated CpG loci that are statistically significant. Gene labels were adjusted using Adobe Illustrator (version 2023) to improve readability. Genes determined to have at least one differentially methylated CpG were then used to perform Gene Ontology (GO) enrichment analysis using the Mouse Genome Informatics’ Visual Annotation Display (VLAD) tool to analyze GO Terms and IDs in differentially methylated genes [43]. A Revigo analysis was then performed to create a TreeMap that summarizes the functional analysis from the enriched GO Terms [44].

DNA methylation in mitochondrial DNA was assessed in the murine model. Features were first limited to only those CpGs that match mitochondrial loci. Differential methylation was calculated as described above and a volcano plot was generated to compare both the MIA and the HIE groups to control. A Circos plot was generated to highlight differential methylation loci on the mitochondrial genome.

To compare results of mitochondrial DNA analysis to maternal immune activation in humans, data were acquired from the Gene Expression Omnibus from a cohort of neonates with histologic chorioamnionitis (HCA) and control cord blood samples [17]. In this data set, methylation data were obtained from cord blood monocytes. After pre-processing and feature pruning to only mitochondrial CpGs, minfi’s dmpFinder was used to identify differentially methylated loci [45].

## 3. Results

### 3.1. MIA Is a Major Contributor to Variation in Gene Methylation Changes in Brain Monocytes

**Figure 1:**
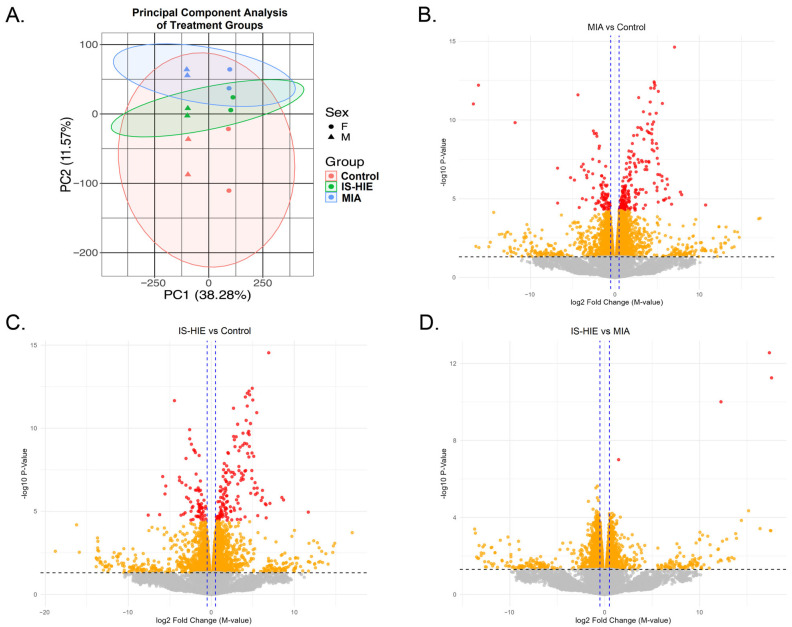
Epigenetic changes after MIA and IS-HIE. (**A**) Principal component analysis demonstrates 49.8% of total variance. PC1 indicates biological variability attributed to sex of the animal (circle, female; triangle, male). PC2 indicates biological variability attributed to exposure group (control, red; IS-HIE, green; MIA, blue). (**B**) Volcano plot demonstrates differential DNA methylation between MIA and control. X-axis indicates a log_2_ fold-change in methylation and the Y-axis; −log_10_(*p*-value). Dashed horizontal line denotes the significance threshold *p* = 0.05. All CpG points are plotted based on the log(*p*) values. Points in grey are non-significant CpGs; orange indicates significant *p*-values; red indicates significant FDR adjusted *p*-values. Dashed vertical lines indicate a threshold for differential methylation by fold-change. (**C**) Volcano plot demonstrates differential DNA methylation between IS-HIE and control. (**D**) Volcano plot demonstrates differential methylation between MIA and IS-HIE.

Differential methylation analysis was performed on brain monocyte isolates collected at postnatal day 7 (P7). Principal component analysis was performed to demonstrate group separation by exposure (Figure 1A). The first principal component (PC1) accounted for 38.3% of the total variance, which clustered by sex (Figure 1B). The second component (PC2) accounted for 11.6% of the total variance, which clustered by exposure group. Combined, a variance of 49.8% was captured across the first two principal components. To characterize the CpG-level differential methylation, we generated Volcano plots to compare MIA vs. control (Figure 1B), and MIA vs. IS-HIE CpGs (Figure 1D). CpG sites were plotted based on the log_2_ fold-change in methylation and statistical significance, with raw *p*-value and FDR-adjusted significance thresholds indicated for comparison. We observed both hypermethylation and hypomethylation with a predominance of hypermethylated CpG sites in the MIA samples (Figure 1C) relative to control. Statistics are available in Supplementary Tables S1–S3.

Comparison of MIA vs. IS-HIE yielded four differentially methylated CpG sites (Figure 1D, Table 1). The UCSC Genome Browser was used to identify the genomic location and associated gene annotations for these CpG sites [46]. Two CpG sites did not map to a gene or known genomic features. One CpG site mapped to two genomic locations represented by *Dph3* and *Oxnad1*. Another CpG site mapped to a candidate cis-regulatory element. Given the lack of significant DNA methylation changes between MIA and IS-HIE, we concluded that most DNA methylation differences in IS-HIE samples were due to the preceding MIA exposure.

### 3.2. Differentially Methylated Genes in MIA Are Involved in Craniofacial Suturing, Development, and Immune Function

**Figure 2:**
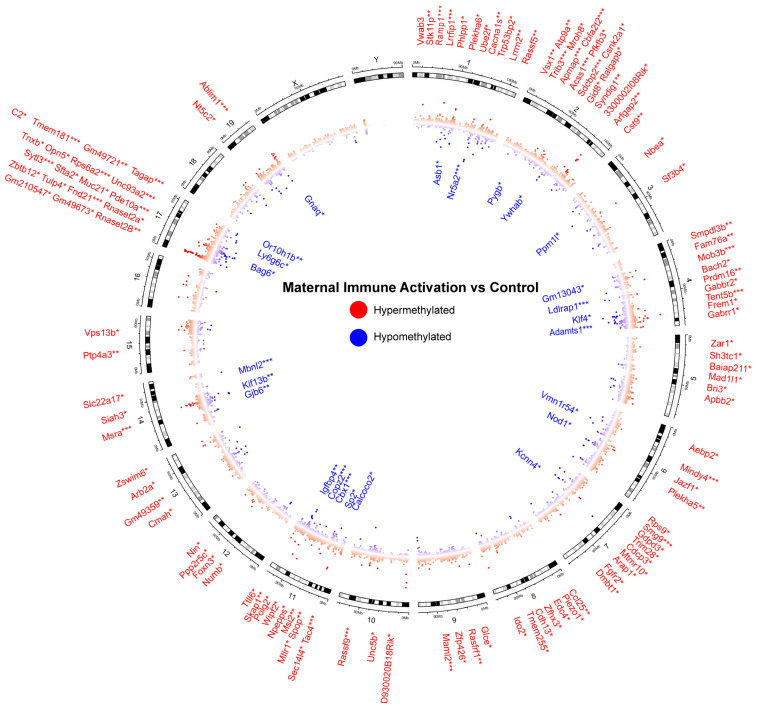
Genomic locations of genes differentially methylated in MIA. Circos plot demonstrating differential DNA methylation across mouse autosomal (1–19) and sex (X and Y) chromosomes. Each point is a CpG methylation score using the mean differential of raw beta-values located at the genomic location. Red denotes hypermethylation and blue denotes hypomethylation in MIA relative to the control. Genes were considered significant if at least one associated CpG site reached FDR-adjusted significance (FDR < 0.05). * denotes FDR adjusted *p*-value of 0.05, ** denotes FDR adjusted *p*-value of 0.01, *** denotes FDR adjusted *p*-value of 0.001.

Differential methylation between control and MIA was visualized with a Circos plot generated to assess the broad distribution of CpG site methylation, restricted to Autosomal and Sex chromosomes (Figure 2). Genes were classified as differentially methylated if there was at least one or more associated CpG sites which reached FDR-adjusted significance (FDR < 0.05). Using this criterion, a total of 140 genes were identified in the MIA vs. Control comparison including 24 hypomethylated and 116 hypermethylated genes. (Supplementary Table S4). A considerable number of enriched genes were located on chromosomes 2 (18 genes), 4 (15 genes), and 17 (21 genes).

**Figure 3:**
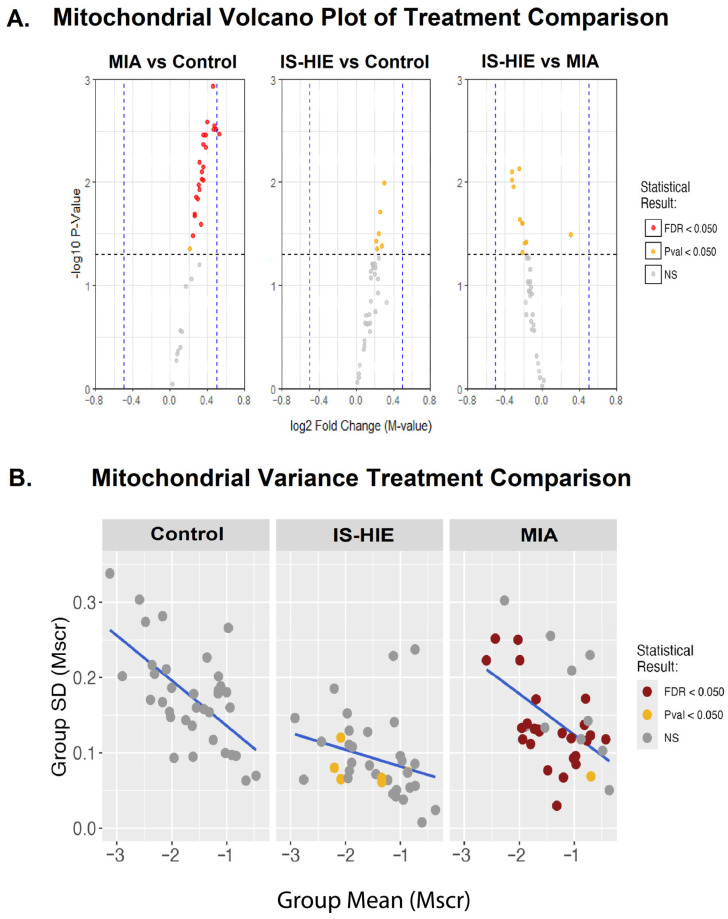
Mitochondrial DNA methylation changes after MIA and HIE. (**A**) Volcano plot demonstrates differential DNA methylation between comparisons of exposure groups in the mitochondrial CpG sites. X-axis indicates a log_2_ fold-change in methylation and the Y-axis; −log_10_(*p*-value). Dashed horizontal line denotes the significance threshold −log_10_(*p* = 0.05). All CpG points are plotted based on the log(*p*) values. Points in grey are non-significant CpGs; orange indicates significant *p*-values; red indicates significant FDR adjusted *p*-values. Dashed vertical lines indicate a threshold for differential methylation by fold-change. (**B**) Plot of variance across mitochondrial CpG sites in each exposure group.

To assess functional enrichment among the genes identified in MIA vs. Control, a Gene Ontology (GO) enrichment analysis was performed using the Mouse Genome Informatics Visual Annotation Display (VLAD) tool. GO biological process terms associated with FDR-significant genes were summarized using the REVIGO TreeMaps function to visualize functional relationships (Table 1). Using a significance threshold of FDR *p* < 0.05, enriched GO terms clustered into distinct functional themes, with prominent representation of regulation of cellular processes and craniofacial suturing pathways (Table 2). To explore broader functional trends, we also conducted REVIGO analysis using a threshold of *p* ≤ 0.1 to capture additional enriched GO terms (Figure 3). All GO Term descriptions and Genes are reported in Supplementary Table S5.

Differentially methylated genes associated with immune signaling, transcriptional regulation, neurodevelopment, and morphogenesis were identified in the MIA vs. control comparison. Among those genes related to immune function and inflammatory signaling we found differential methylation in *Ido2*, which has been implicated in microglial activation and seizures [47]; *Lrrfip1*, a nuclear regulator of TNF and innate immune system protein involved in wound repair and oncogenesis [48,49]; *Phlpp1*, a known counter-regulator of STAT1-mediated inflammatory signaling [50]; *Kcnn4*, a critical mediator of T cell activation and microglia migration [51]; and *Ccl25*, a chemokine involved in TH17 response in multiple organ systems [52,53]. *Foxn3* and *MsrA* were also differentially methylated in MIA. *Foxn3* regulates NF-kB transcriptional activity and ameliorates *MsrA*-associated oxidative stress responses [54]. *MsrA* exerts a neuroprotective influence on embryonic stem cells against ischemic and reperfusion stress by reversing oxidized proteins to their functional configuration [55].

Several transcription factors and transcriptional regulators that are critical to neurodevelopment were also identified such as *Klf4*, a transcription factor essential for neural differentiation and linked to hydrocephalus when dysregulated [56]; *Bach2*, an oxidative stress regulating transcription factor for neuronal cell survival [57]; and *Zfhx3*, important for neurodevelopment and cell differentiation [58]. Also identified were *Trim28*, which regulates transposable elements in the developing brain [59]; *Prdm16*, required for neural stem and progenitor cell differentiation [60]; *Aebp2*, a transcriptional regulator of neural crest cell development [61]; and *Sp2*, a transcriptional regulator for a myriad of critical cellular processes [62].

Genes associated with developmental processes and structural organization were also identified. These include *Numb*, which is involved in asymmetric cell division and cell fate determination [63]; *Fgfr2*, critical for cranial skeleton development and wound healing [64]; *Pfkfb3*, a potential therapeutic target for cerebral ischemia-reperfusion injury given its role in regeneration of NADPH [65]; and *Frem1*, essential for extracellular matrix organization and basement membrane structure, therefore, associated with multiple congenital malformations [66]. *Polg2*, critical for mammalian embryogenesis and mitochondrial DNA replication, was identified as differentially methylated [67].

We also found that Nr5a2 was hypomethylated in MIA. Polymorphisms in this gene are associated with increased risk of premature delivery, a known risk factor for adverse neurologic outcomes [66]. This is particularly interesting given the significantly increased risk of premature delivery in pregnancies complicated by chorioamnionitis [68].

### 3.3. Mitochondrial DNA Exhibits Consistent Hypermethylation Following Inflammatory Insult

To investigate methylation changes that may influence mitochondrial function, we analyzed mitochondrial CpG methylation differences in MIA compared to control. Notably, 25 of 36 identified mitochondrial CpG sites had statistically significant values of differential methylation in MIA, all of which were hypermethylated in MIA relative to control (Figure 3A, Table 2). IS-HIE also demonstrates a trend toward hypermethylation relative to control, although no CpG loci are statistically differentially methylated after false discovery rate correction (Figure 3A). MIA and IS-HIE mitochondrial DNA methylation did not have statistically significant differences after FDR correction (Figure 3A). Due to the contrast between MIA and IS-HIE mitochondrial methylation, we assessed variance of methylation in the mitochondrial CpGs in control, MIA, and IS-HIE (Figure 3B). Interestingly, variance is highest in control samples but is similar between control and MIA. In IS-HIE, however, overall variance is reduced, suggesting the possibility of selection bias after exposure to hypoxia.

To determine whether mitochondrial CpGs are differentially methylated in human maternal immune activation exposure, we used previously reported neonatal human cord blood histologic chorioamnionitis data [17] available in the gene expression omnibus (GEO accession number GSE153668). Data were pre-processed and filtered to include only mitochondrial DNA, using a custom manifest with mitochondrial DNA [45]. Statistically significant hypermethylation was identified in one CpG (exon 1 of *mt-CO1*) of twelve that were identifiable in the dataset. Of note, *mt-CO1* was not among the differentially methylated genes found in mouse MIA (Table 3).

## 4. Discussion

MIA is a significant risk factor for adverse neurodevelopmental outcomes, particularly in combination with HIE [8,9,13,14,15,16]. In this study, we demonstrate that MIA induces widespread DNA methylation differences in brain monocytes. We identified differentially methylated transcription factors that mediate neurodevelopment, providing a potential mechanism for the increased neurodevelopmental risk imparted by MIA. We also identified differentially methylated genes involved in immunological function, inflammatory signaling, transcriptional regulation, and neurodevelopmental processes. Overall, we found that MIA exerts a considerable influence on the neonatal epigenetic profile in brain monocytes.

Principal component analysis (Figure 1A) demonstrates that MIA and IS-HIE samples overlap, while both are distinct from the control samples, suggesting that DNA methylation changes in HIE and MIA are similar. We observed minimal variation between DNA methylation patterns in MIA and HIE, with only 4 significant CpGs, only one of which is associated with the coding region of a gene. The limited number of differentially methylated CpGs in the IS-HIE vs. MIA comparison, combined with only a single CpG site per gene raises concerns about the biological validity of the differentially methylated loci we identified in IS-HIE. However, the small sample size in our study may limit the generalizability of this finding. In total, we identified 140 differentially methylated nuclear genes in the MIA vs. control comparison, containing at least one differentially methylated CpG with an FDR-adjusted *p*-value significance less than 0.05. Of these, 116 genes were hypermethylated, while 24 genes were hypomethylated.

While investigating the comparisons between IS-HIE and MIA treatments, only four hypermethylated CpG loci were reported to be significant with FDR-corrected *p*-values. To assess the biological relevance of these four CpG sites, we used the UCSC Genome Browser to annotate genomic features. Notably, Cg41388844 (two probes) did not map any known annotations in mm10. Cg40977002 was localized at the site of two overlapping genes; *Dph3* at the Exon 1 coding region and *Oxnad1* at the 5′ untranslated region. *Dph3* is essential for diphthamide synthesis which modifies elongation factor 2 *(EEF2)*, a key post-translational modification involved in translational integrity. Although not directly associated with this CpG site, *Fam76a*-reported in our MIA vs. Control gene list and not associated with this CpG loci- may also regulate the ratio of *Dph3* transcript in response to intermittent hypoxia [69]. *Dph3* is also reported to be regulated by parental noncoding RNA molecules with downstream regulation in oocytes [69]. *Oxnad1* is involved in oxidoreductase activity. Hypermethylation may enhance or reduce transcription or exon usage, potentially impacting cell growth (mTOR) pathways [70] and maternal-fetal cellular stress response [71]. Furthermore, Cg40977002 is a predicted candidate for cCRE and likely involved in learning and memory, chromatin organization, and transcriptional regulation [72]. CTCF-mediation and enhancer promoter interactions are critical for genome organization and can cause a variety of developmental disorders with intellectual disabilities [73].

Gene Ontology analysis reveals possible mechanisms underlying the increased neurologic risk imparted by maternal immune activation in utero. This analysis highlighted immunological functioning changes, increased inflammatory signaling, stress response signaling, programmed cell death and differentiation, neurodevelopmental and morphological processes. Inflammatory and immunological gene regulation fit with the primary functions of brain monocytes, which include intrinsic microglia and infiltrating macrophages. These monocytes are the primary initial cellular responders to brain injury or inflammation. Alterations in inflammatory and immunological gene regulation after MIA may alter the response of brain monocytes to exacerbate neuronal or glial phagocytosis, resulting in poorer long term functional outcome. However, additional studies are needed to definitively link these methylation changes to gene transcriptional differences and functional monocyte changes in response to MIA. The significant number of transcriptional regulators that demonstrate altered methylation after MIA is notable, given the possibility of broad downstream gene regulation changes conveyed by altered expression of transcription factors.

Surprisingly, we identified statistically significant hypermethylation in the majority of CpG sites in the mitochondrial genome after MIA exposure. Previous studies have shown that differential methylation in mitochondrial DNA regulates mitochondrial gene expression and cellular metabolism, crucial for neurodevelopment and brain function [74,75]. Our findings suggest that MIA may impact mitochondrial gene expression and may increase the risk of failure of oxidative phosphorylation. Notably, IS-HIE mitochondria did not demonstrate the same degree of statistically significant difference seen in MIA mitochondria. Variance analysis reveals that IS-HIE mitochondrial methylation has significantly lower variance compared to control and MIA mitochondria CpG sites. This raises the possibility that mitochondria are under selection pressure conferred by the combined exposure to MIA and subsequent hypoxia that results in loss of metabolically “at risk” mitochondria or increased biogenesis of metabolically stable mitochondria. Analysis of previously published human data from cord blood samples from neonates with chorioamnionitis exposure also identified hypermethylation in mitochondrial DNA, however, the limited number of patients included in this analysis and limitations of detection of mitochondrial CpGs using Illumina sequencing likely limit definitive conclusions that can be drawn from this data. However, this data suggests that mitochondrial DNA methylation may be an important and clinically relevant direction for future research into the mechanisms of neurologic risk conveyed by MIA in humans.

One limitation of our study is the selective focus on brain monocytes which limits our conclusions regarding widespread DNA methylation changes to the immune cells within the brain. However, brain monocytes are the primary responders to brain insult or injury and play a key role in dictating the response and remodeling of the injured tissue [21,76]. Future studies investigating DNA methylation changes in neural or glial cells will help determine whether these effects are unique to the brain monocyte population or are more broadly shared among brain cell types. Another potential limitation is the absence of a hypoxia exposure group to confirm whether hypoxia alone induces DNA methylation. However, hypoxia was intentionally omitted in this study as our primary goal was to investigate the mechanisms of neural risk conveyed by MIA. The small sample size in this study, with four biological replicates per group, likely limits identification of smaller variations in DNA methylation. Statistical limitations are also likely given that biological replicates were siblings with similar in utero exposures. Given the small sample size, we did not analyze sex effects on DNA methylation differences between groups. Future studies can use this data for sample size estimation to determine both small and large effects.

## 5. Conclusions

Our findings demonstrate widespread DNA methylation changes in brain monocytes after MIA or IS-HIE in genes that are important for immune activation, neurodevelopment, craniofacial development, and mitochondrial function, underscoring the need for future investigation into the transcriptional and functional mechanisms conveyed by these methylation differences.

## Figures and Tables

**Table 1:** Biological Features of CpG probes differentially methylated in IS-HIE and not in MIA.

CpG	Genomic Location	Biological Feature
cg41388844 (2 probes)	Chromosome 4: 124,110,885–124,110,886	Unannotated in mm10 (GRC38)
cg40977002	Chromosome 14: 32,085,502–32,085,503	Exon 1 (coding region) of *Dph3* 5′ Untranslated region of *Oxnad1*
cg40977002	Chromosome 4: 63,622,662–63,622,663	Candidate for cis-regulatory elements (cCRE); predicted in pELS/CTCF-bound site

**Table 2:** Biological processes of differentially methylated genes after MIA exposure.

GO Term ID	Term Description	FDR-Adjusted *p*-Value	Gene Symbols
GO:0051253	Negative regulation of RNA metabolic process	4.30 × 10^−2^	*Aebp2*, *Apbb2*, *Bach2*, *Cbfa2t2*, *Cbx1*, *Fgfr2*, *Foxn3*, *Gm13043*, *Jazf1*, *Klf4*, *Lrrfip1*, *Prdm16*, *Sp2*, *Spop*, *Tent5b*, *Trib3*, *Trim28*, *Ywhab*, *Zar1*, *Zbtb12*, *Zfhx3*
GO:0045934	Negative regulation of nucleobase-containing compound metabolic process	4.30 × 10^−2^	*Aebp2*, *Apbb2*, *Bach2*, *Cbfa2t2*, *Cbx1*, *Csnk2a1*, *Fgfr2*, *Foxn3*, *Gm13043*, *Jazf1*, *Klf4*, *Lrrfip1*, *Prdm16*, *Sp2*, *Spop*, *Tent5b*, *Trib3*, *Trim28*, *Ywhab*, *Zar1*, *Zbtb12*, *Zfhx3*
GO:0097094	Craniofacial suture morphogenesis	4.65 × 10^−2^	*Fgfr2*, *Foxn3*, *Frem1*
GO:0045892	Negative regulation of DNA-templated transcription	4.65 × 10^−2^	*Aebp2*, *Apbb2*, *Bach2*, *Cbfa2t2*, *Cbx1*, *Fgfr2*, *Foxn3*, *Gm13043*, *Jazf1*, *Klf4*, *Lrrfip1*, *Prdm16*, *Sp2*, *Spop*, *Trib3*, *Trim28*, *Ywhab*, *Zbtb12*, *Zfhx3*
GO:1902679	Negative regulation of RNA biosynthetic process	4.65 × 10^−2^	*Aebp2*, *Apbb2*, *Bach2*, *Cbfa2t2*, *Cbx1*, *Fgfr2*, *Foxn3*, *Gm13043*, *Jazf1*, *Klf4*, *Lrrfip1*, *Prdm16*, *Sp2*, *Spop*, *Trib3*, *Trim28*, *Ywhab*, *Zbtb12*, *Zfhx3*
GO:0050794	Regulation of cellular process	4.65 × 10^−2^	*3300002I08Rik*, *Ablim1*, *Aebp2*, *Apbb2*, *Arap1*, *Arb2a*, *Asb1*, *Atp9a*, *Bach2*, *Bag6*, *Baiap2l1*, *C2*, *Cacna1s*, *Calcoco2*, *Cbfa2t2*, *Cbx1*, *Ccl25*, *Cdh13*, *Cmah*, *Csnk2a1*, *Dmbt1*, *Edc4*, *Fgfr2*, *Fndc1*, *Foxn3*, *Gabbr2*, *Gabrr1*, *Gid8*, *Gjb6*, *Glce*, *Gm13043*, *Gm49359*, *Gnaq*, *Igfbp4*, *Jazf1*, *Kcnn4*, *Kif13b*, *Klf4*, *Ldlrap1*, *Lrrfip1*, *Mad1l1*, *Maml2*, *Mbnl2*, *Milr1*, *Mob3b*, *Msi2*, *Muc21*, *Nbea*, *Nin*, *Nod1*, *Nr5a2*, *Numb*, *Opn5*, *Or10h1b*, *Pde10a*, *Phlpp1*, *Piezo1*, *Polg2*, *Ppm1l*, *Ppp2r5c*, *Prdm16*, *Ptp4a3*, *Ralgapb*, *Ramp1*, *Rasgrf1*, *Rassf5*, *Rassf9*, *Rps6ka2*, *Skap1*, *Smg9*, *Smpdl3b*, *Sp2*, *Spop*, *Syndig1*, *Tac4*, *Tagap*, *Tent5b*, *Tnxb*, *Trib3*, *Trim28*, *Ttll6*, *Unc5b*, *Vmn1r54*, *Vsx1*, *Ywhab*, *Zar1*, *Zbtb12*, *Zfhx3*, *Zswim6*
GO:0048519	Negative regulation of biological process	4.65 × 10^−2^	*Aebp2*, *Apbb2*, *Arap1*, *Arb2a*, *Atp9a*, *Bach2*, *Bag6*, *Cacna1s*, *Cbfa2t2*, *Cbx1*, *Ccl25*, *Cdh13*, *Csnk2a1*, *Edc4*, *Fgfr2*, *Foxn3*, *Gjb6*, *Glce*, *Gm13043*, *Gnaq*, *Jazf1*, *Kcnn4*, *Klf4*, *Lrrfip1*, *Mad1l1*, *Milr1*, *Muc21*, *Nin*, *Nr5a2*, *Nt5c2*, *Numb*, *Pde10a*, *Phlpp1*, *Ppp2r5c*, *Prdm16*, *Rassf5*, *Rps6ka2*, *Siah3*, *Smg9*, *Smpdl3b*, *Sp2*, *Spop*, *Tac4*, *Tent5b*, *Trib3*, *Trim28*, *Unc5b*, *Ywhab*, *Zar1*, *Zbtb12*, *Zfhx3*
GO:0050789	Regulation of biological process	4.65 × 10^−2^	*3300002I08Rik*, *Ablim1*, *Aebp2*, *Apbb2*, *Arap1*, *Arb2a*, *Asb1*, *Atp9a*, *Bach2*, *Bag6*, *Baiap2l1*, *C2*, *Cacna1s*, *Calcoco2*, *Cbfa2t2*, *Cbx1*, *Ccl25*, *Cdh13*, *Cmah*, *Csnk2a1*, *Dmbt1*, *Edc4*, *Fgfr2*, *Fndc1*, *Foxn3*, *Gabbr2*, *Gabrr1*, *Gid8*, *Gjb6*, *Glce*, *Gm13043*, *Gm49359*, *Gnaq*, *Igfbp4*, *Jazf1*, *Kcnn4*, *Kif13b*, *Klf4*, *Ldlrap1*, *Lrrfip1*, *Mad1l1*, *Maml2*, *Mbnl2*, *Milr1*, *Mob3b*, *Msi2*, *Muc21*, *Nbea*, *Nin*, *Nod1*, *Nr5a2*, *Nt5c2*, *Numb*, *Opn5*, *Or10h1b*, *Pde10a*, *Phlpp1*, *Piezo1*, *Polg2*, *Ppm1l*, *Ppp2r5c*, *Prdm16*, *Ptp4a3*, *Ralgapb*, *Ramp1*, *Rasgrf1*, *Rassf5*, *Rassf9*, *Rps6ka2*, *Siah3*, *Skap1*, *Smg9*, *Smpdl3b*, *Sp2*, *Spop*, *Syndig1*, *Tac4*, *Tagap*, *Tent5b*, *Tnxb*, *Trib3*, *Trim28*, *Ttll6*, *Unc5b*, *Vmn1r54*, *Vsx1*, *Ywhab*, *Zar1*, *Zbtb12*, *Zfhx3*, *Zswim6*

**Table 3:** Differentially methylated mitochondrial genes after MIA exposure.

Gene	FDR-Adjusted *p*-Value	Direction of Change
*Rnr-1*	1.26 × 10^−2^	Hypermethylated (MIA > Control)
*Rnr-2*	1.26 × 10^−2^	Hypermethylated (MIA > Control)
*Nd-1*	1.26 × 10^−2^	Hypermethylated (MIA > Control)
*Tm*	1.26 × 10^−2^	Hypermethylated (MIA > Control)
*Nd-2*	2.51 × 10^−2^	Hypermethylated (MIA > Control)
*Nd-4*	1.26 × 10^−2^	Hypermethylated (MIA > Control)
*Nd5*	1.90 × 10^−2^	Hypermethylated (MIA > Control)
*CytB*	1.26 × 10^−2^	Hypermethylated (MIA > Control)

## Data Availability

The data presented in this study are openly available in [NCBI GEO accession number GSE319589].

## Supplementary Materials

The following supporting information can be downloaded at: https://www.mdpi.com/article/10.3390/cells15080714/s1, Table S1: MIA vs. Control Differential Methylation and Log Fold Change statistics. Table S2: HIE vs. Control Differential Methylation and Log Fold Change statistics. Table S3: HIE vs. MIA Differential Methylation and Log Fold Change statistics. Table S4: Methylated Gene Table: MIA vs. Control; Table S5: Revigo Plot GO Term Associations.

## Abbreviations

The following abbreviations are used in this manuscript:

FDR	False discovery rate
GO	Gene ontology
HIE	Hypoxic ischemic encephalopathy
LPS	Lipopolysaccharide
IS-HIE	Inflammation sensitized HIE
MIA	Maternal immune activation
PCA	Principal component analysis
